# High-density EEG mobile brain/body imaging data recorded during a challenging auditory gait pacing task

**DOI:** 10.1038/s41597-019-0223-2

**Published:** 2019-10-17

**Authors:** Johanna Wagner, Ramon Martinez-Cancino, Arnaud Delorme, Scott Makeig, Teodoro Solis-Escalante, Christa Neuper, Gernot Mueller-Putz

**Affiliations:** 1 Swartz Center for Computational Neuroscience, Institute for Neural Computation, University of California San Diego, La Jolla, CA USA; 2 Electric and Computer Engineering Department, Jacobs School of Engineering, University of California San Diego, La Jolla, CA USA; 3 Laboratory for Brain Computer Interfaces, Institute of Neural Engineering, Graz University of Technology, Graz, Austria; 4 Department of Psychology, University of Graz, Graz, Austria; 5 Department of Rehabilitation, Donders Institute for Brain, Cognition and Behavior, Radboud University Medical Center, Nijmegen, The Netherlands

**Keywords:** Cognitive control, Sensorimotor processing, Electroencephalography - EEG

## Abstract

In this report we present a mobile brain/body imaging (MoBI) dataset that allows study of source-resolved cortical dynamics supporting coordinated gait movements in a rhythmic auditory cueing paradigm. Use of an auditory pacing stimulus stream has been recommended to identify deficits and treat gait impairments in neurologic populations. Here, the rhythmic cueing paradigm required healthy young participants to walk on a treadmill (constant speed) while attempting to maintain step synchrony with an auditory pacing stream and to adapt their step length and rate to unanticipated shifts in tempo of the pacing stimuli (e.g., sudden shifts to a faster or slower tempo). High-density electroencephalography (EEG, 108 channels), surface electromyography (EMG, bilateral tibialis anterior), pressure sensors on the heel (to register timing of heel strikes), and goniometers (knee, hip, and ankle joint angles) were concurrently recorded in 20 participants. The data is provided in the Brain Imaging Data Structure (BIDS) format to promote data sharing and reuse, and allow the inclusion of the data into fully automated data analysis workflows.

## Abstract (summary)

**Table:** 

Measurement(s)	brain activity measurement • electric potential • heel strike • joint angle • auditory stimulation
Technology Type(s)	electroencephalography (EEG) • electromyography • Pressure Sensor Device • goniometer • computer
Sample Characteristic - Organism	Homo sapiens

Machine-accessible metadata file describing the reported data: 10.6084/m9.figshare.9919376

## Background & Summary

The ability to modify an initiated step in response to environmental challenges is often impaired in the elderly and individuals with neurologic gait pathologies such as stroke and Parkinson’s disease^1,2^. The impaired ability to successfully adapt the gait pattern^3,4^ reduces the ability to avoid obstacles and increases the risk of falling^5^, which is a major reason for morbidity and disability in these populations^6^. One salient feature of these neurologic gait impairments, is a decrease in the ability to regulate stride-to-stride fluctuations and an increase in gait variability^7–9^, which has been related to a decline in executive function^9,10^.

A recommended method to identify and treat gait adaptation deficits is the use of an auditory pacing stream^11–15^. Temporally predictable auditory cues have beneficial effects on gait by increasing speed, stride length, and improving symmetry and stability^16,17^; and these benefits can generalize to non-cued gait after extensive periods of training^18–21^. To coordinate steps to the rate and phase of an auditory pacing stream, individuals must be able to extract the beat from the auditory sequence, match their gait cadence to the stimulus rate, and time goal-directed movements to beat onsets, factors which may be crucial to predicting the success of rhythmic auditory training for affected individuals.

Unfortunately, because of the ill effects of body movements on brain imaging data, the precise temporal brain dynamics of gait adaptation to rhythmic auditory cueing remain largely unexplored. Recent signal processing advances, however, allow studying source-resolved electroencephalography (EEG) dynamics during walking^22–26^ and other actions, an approach termed Mobile Brain/Body Imaging (MoBI)^27^.

In this report, we present a multimodal dataset from 20 healthy young participants that allows to study coordination of steps to the timing and rate of the auditory pacing stream as well as executive function in gait adaptation. Participants were instrumented with high-density EEG (108 channels), surface electromyography (EMG) with electrodes placed on the tibialis anterior muscle of both legs, pressure sensors on the heel to measure heel strikes, and goniometers measuring joint angles of ankle, knee, and hip. Participants walked on a treadmill at a constant speed while attempting to step in synchrony with an auditory pacing stream and were required to adapt their step length and rate to shifts in tempo of the pacing stimulus (e.g., to unexpected shifts to a faster or slower pacing tempo). To our knowledge this is the first published dataset featuring EEG recordings during a dynamic gait adaptation task requiring synchronization of steps to auditory cues and one of only three other public EEG-MOBI datasets recorded during walking^28–30^.

We have used this dataset to investigate top-down inhibitory control in gait adaptation modeling source-resolved oscillatory cortical dynamics and event-related potentials time locked to cue tones and heel strikes following cue rate shifts^31,32^. These data could be used to support further studies of gait adaptation, error processing, and auditory-motor synchronization during walking, analyses that might give further insights into the underlying cortical mechanisms of auditory rhythmic cue training. This has significant importance for the field of gait rehabilitation in the elderly and Parkinson’s disease^12–15^. The multimodal nature of the dataset allows for investigation of relationships between EEG and EMG during walking, including corticomuscular coherence, and joint analysis of movement parameters and EEG. The dataset contains data from 20 subjects, 18 of which have sufficiently clean EEG data for meaningful analysis, and 16 have kinematic data (not crucial for EEG analysis since heel strike markers come from pressure sensors, whose records are present for all subjects). The number of subjects in our study is relatively high compared to other EEG studies of walking with fewer subjects, in which significant effects have been demonstrated. These include studies using fewer than 15 participants that have demonstrated significant power modulations relative to the gait cycle^23,25,26,33,34^ and significant effects of visual feedback during walking^33^. Other studies have demonstrated corticomuscular coherence during walking using fewer than 12 participants^35,36^ and significant relationships between EEG and kinematics in only 6 subjects^37^. We therefore believe that our data set is suited to addressing many questions concerning the EEG brain dynamics that accompany cue-paced gait and gait adjustment.

## Methods

### Participants

Twenty healthy volunteers (9 females and 11 males, 22–35 years of age; mean 29.1 years, SD 2.7 years) with no neurological or motor deficits participated in this study. The EEG data of two subjects (participant IDs 19 and 20) was heavily contaminated by artifacts and was therefore excluded from the analysis for data validation. Nonetheless we provide the data of these two subjects for download and tag them as noisy since they might be useful for people developing tools for artifact removal. All participants reported being right handed. Research shows that footedness follows handedness in right handers, although not consistently so in left handers^38^. The experimental procedures were approved by the human ethics committee of the Medical University Graz, Austria. Each subject gave signed informed consent before the experiment.

### Data acquisition

**Fig. 1:**
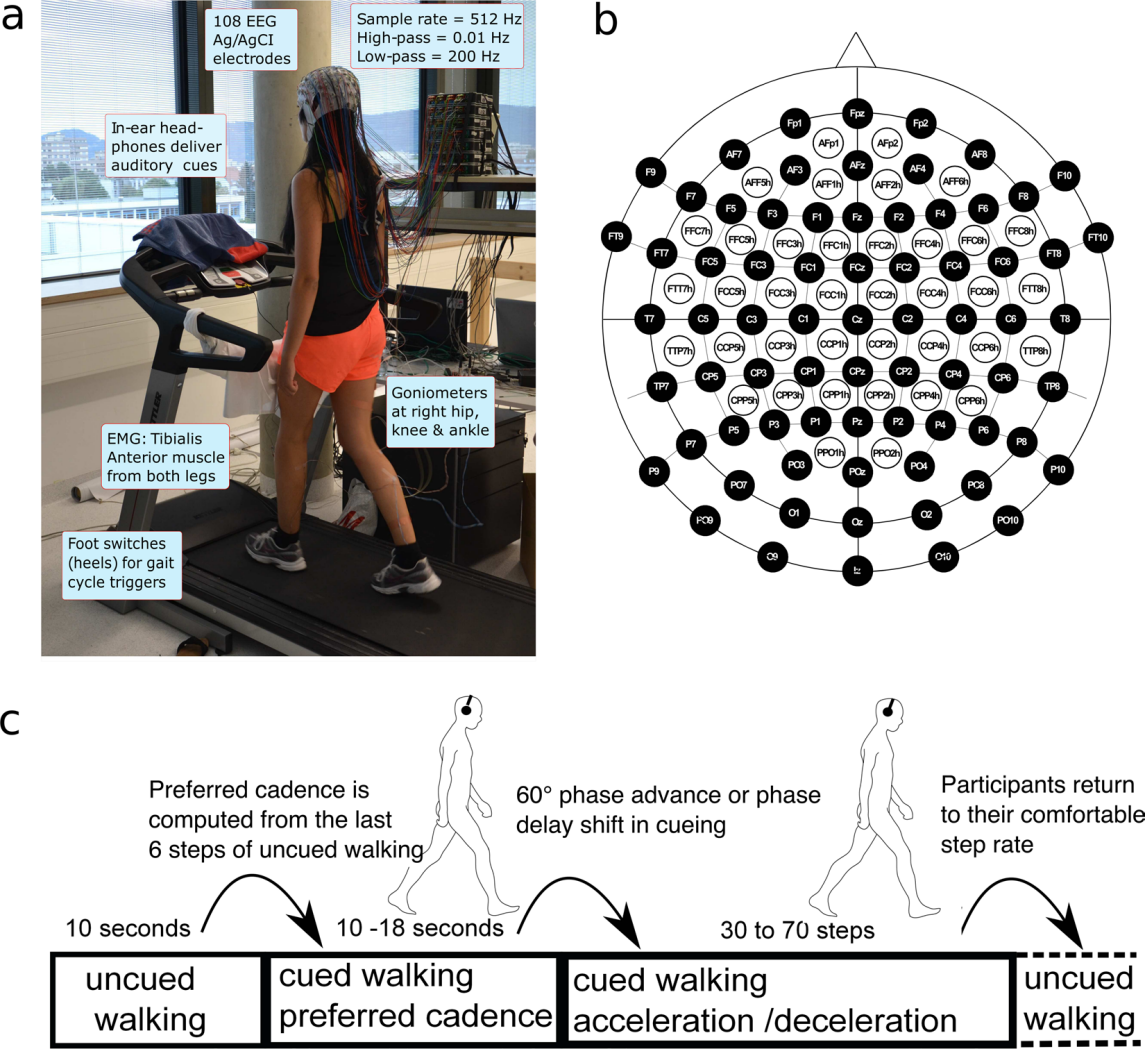
Experimental setup and paradigm. (**a**) Experimental setup. Participant walking on the treadmill with auditory pacing cues delivered through in-ear headphones. During the initial training period, treadmill speed (3–3.7 km/h) was adjusted to the most comfortable pace for each participant and thereafter remained constant. (**b**) Electrode layout. We recorded 108 EEG channels placed according to the 10 per cent system. Reference and ground electrode were placed on left and right mastoid. (**c**) Treadmill speed was adapted and fixed at a comfortable walking speed by the participant and remained fixed throughout the experiment. During each trial, participants first walked for ~10 s without auditory cues, then walked for 10–18 s while attempting to synchronize their foot falls to brief cue tones delivered at their then-prevailing step rate and phase. Thereafter, beginning at a right heel strike, a sudden (accelerated or decelerated) tempo shift occurred in the pacing cue sequence. In response, participants were instructed to adapt their step length, rate, and phase as quickly as possible, so as to again synchronize their steps with the cue tones at the new tempo. After 30–70 steps, the next trial began immediately, returning again to 10 s of uncued walking during which participants were instructed to return to their most comfortable step rate. The tempo shift always occurred relative to a right step, the first deviant tone indicating the new tempo by being early (in step-advance trials) or late (e.g., in step-delay trials). Figure adapted from^31,32^.

The recordings were performed at the Institute of Neural Engineering at Graz University of Technology, Austria. Seven 16-channel amplifiers (g.tec GmbH, Graz, Austria) were combined to record EEG data from 108 passive scalp electrodes positioned as in the ‘5% International 10/20 System’ (EasyCap, Herrsching, Germany)^39^. Each subject’s head circumference was measured to allow for selection of an appropriately sized EEG cap. The cap was aligned on the head such that Cz was 50% of the distance from the nasion to the inion along the midsagittal plane and 50% of the distance from preauricular points. Electrode locations that extended below the conventional 10–20 System layout included F9, FT9, F10, FT10, P9, PO9, P10, PO10, I1, Iz, I2 (for a schema of the electrode layout see Fig. 1b). Reference and ground electrodes were placed on the left and right mastoids, respectively. All EEG electrode impedances were brought below 5 kΩ before recording.

Electromyographic (EMG) signals were recorded from the skin over the tibialis anterior muscles of both legs using standard adhesive-fixed disposable Ag/AgCl surface electrodes. These EMG channels were also recorded using left and right mastoids as reference and ground, respectively. The EEG and EMG data were sampled at 512 Hz, high pass filtered >0.1 Hz, low pass filtered <256 Hz and a notch filter was applied at 50 Hz to remove power line noise.

Foot-ground contacts were measured by mechanical foot switches placed over the calcaneus bone in the heel of each foot. These switches produced event markers for gait cycle heel strike and heel off events. We also recorded data from three flexible segment twin axis goniometers placed on the right hip, knee and ankle (Plux, Wireless Biosignals, Arruda dos Vinhos, Portugal). The goniometers were attached to the body using medical adhesive tape and placed such that the center of the goniometer was over the joint on the right side of the leg (compare to^29^).

The data were recorded with the TOBI SignalServer, a custom software application for data acquisition developed at the Institute of Neural Engineering at Graz University of Technology^40,41^, and a Simulink script running on MATLAB 2013 (The MathWorks). The TOBI SignalServer, a cross-platform data acquisition system implemented in C++, is designed to support concurrent multirate acquisition from different hardware devices with a focus on performance and stability. The code for the TOBI signal server can be obtained from *tools4bci.github.io/SignalServer*. In each trial the Simulink model calculated step-by-step cadence (time interval between heel strikes) and adjusted the pacing of the auditory stimulus in the preferred (steady-state) walking condition using the mean of the most recent 6 steps of uncued walking. We also classified left versus right heel strikes in the Simulink model to make sure that the first auditory cue in each trial, as well as the cues marking the tempo changes always occurred relative to a right heel strike. An application in Ruby (https://www.ruby-lang.org/en/) was developed to play the auditory cues.

To record the exact timing of the auditory cues, we recorded the auditory stimulation as a digital input to one of the amplifiers. To this purpose we split the auditory output of the computer so as to feed the auditory cues into a digital circuit that amplified the analog signal so as to drive a transistor into saturation, thus providing a 5-V (TTL) signal to be directly connected to a digital input of the EEG amplifier. All triggers were synchronized via the hardware input of the amplifiers. The goniometers were synchronized to the EEG data using the TOBI SignalServer.

During the recording we monitored the EEG data for possible large muscle and movement artifacts. We instructed the participants to relax their shoulders to avoid neck muscle artifacts and to move their head as little as possible. The amplifiers were placed next to the treadmill on two stacked tables so that they were close to the participant’s head.

### Experimental design and procedure

#### Trial structure

During the experiment participants walked on the treadmill and were instructed to synchronize their steps to a regular auditory pacing stream into which were introduced infrequent sudden shifts to a slower or faster tempo. Participants were asked to adapt their steps as quickly as possible to the new tempo so as to synchronize with the auditory cues. In each trial (Fig. 1), participants walked at their self-selected comfortable pace without auditory cueing for 10 s, after which a stream of auditory cue tones was delivered at their current step tempo. To make it easier for participants to synchronize with the auditory cue stream, the first tempo-shifted cue onset was always close to a right heel strike. The tempo of the auditory cue tones was computed as the mean heel strike interval (heel strike to heel strike) across their 6 most recent non-cued steps. This was done to ensure that auditory cues always matched their current comfortable walking speed, which slightly varies across trials. Auditory cue tones were delivered via in-ear headphones. The cue sequence was an alternating series of high and low tones presented so as to allow a match to the participant’s alternating right and left heel strikes; high/low tone assignment to left/right or right/left steps, respectively, was randomized over subjects (the auditory tones were 100 ms in duration; low tones were sinusoids at 325 Hz, high tones at 512 Hz. Auditory tones were generated in MATLAB (The Mathworks), and played as *.wav* files).

Participants were asked to attempt to synchronize their heel strikes to the regular sequence of auditory cue tones, thus building an expectation of when the next cue would occur. After walking 8–12 s to auditory cues at the preferred cadence, the tempo of the cue stream was suddenly increased (‘step-advance’ perturbation) or decreased (‘step-delay’ perturbation) by one-sixth of a step cycle, plus a random ≤±25 ms jitter. The cue tempo shift always occurred relative to a right heel strike (as in^42^). In step-advance or step-delay perturbations, the cue marking the tempo shift would thus seem to participants to arrive either ‘too early’ (in step-advance) or ‘too late’ (step-delay).

Participants were instructed to adjust their steps as quickly as possible in order to synchronize their heel strikes to the tone cues at the new pacing tempo, which was maintained for 30–70 steps (see Fig. 1). Since the treadmill moved at a constant speed throughout the experiment, participants had to implement gait adjustments either by producing (in step-delay trials) one-sixth longer steps, or (in step-advance trials) one-sixth shorter steps.

After 30–70 steps at the new stepping rate, the next trial began immediately, again with uncued walking. Participants were instructed to return to their most comfortable step length and tempo during this period. We conducted a total of 60 step-advance and 60 step-delay trials in 10 blocks of 12 trials. Each block was comprised of 6 step-advance and 6 step-delay trials presented in random order. Between blocks, 5-min breaks were given if asked for by participants. Participants either remained standing or sitting on a chair that we placed on the treadmill during breaks if required. For a picture of the experimental setup and paradigm see Fig. 1.

In addition, we recorded two blocks of four minutes with participants walking on the treadmill without cues for two minutes, we then stopped the treadmill and participants remained standing on the treadmill and listening to auditory cues at the cadence of their previous footfalls. These blocks were randomly interspersed with the task blocks.

#### Training

The experiment used a conventional treadmill (Kettler, Track S4, Ense-Parsit, Germany). Before starting the experimental procedure, we asked participants to walk on the treadmill for 2–3 min to become familiar with treadmill walking. During the subsequent practice period, we asked participants to adapt the belt speed to their most comfortable walking speed, and so determined, for each participant in the experiment, a belt speed that was held fixed throughout the experiment. Walking speed ranged from 3.0 to 3.7 km per hour between participants, lower than the typically reported comfortable overground walking speed (near 4.6 km/h for women and 5.2 km/h for men^43^). To familiarize themselves with the task, participants then practiced walking on the treadmill for about 5 min while attempting to step in synchrony to an auditory cue tone stream. Before beginning the experiment we made sure the participant understood the walking task and was able to perform the gait synchronization task to an acceptable performance level, meaning that participants lengthened or shortened their steps appropriately to adapt their steps to cue advance or delay tempo shifts in the auditory cue sequence, so as to synchronize to the new pacing tempo.

## Data Records

All the published data sets are de-identified. All data files are available at *OpenNeuro.org* with accession number ds001971^44^, organized and archived following the EEG-Brain Imaging Data Structure (BIDS)^45,46^. The study was converted to EEG-BIDS using the EEGLAB-to-BIDS plug-in by Delorme & Pernet (*github.com/sccn/bids-matlab-tools*) for EEGLAB^47^, running on MATLAB (The MathWorks). The BIDS specification^45^ is a human brain research community standard for organizing and sharing brain imaging data within and between laboratories which has become widely used for archiving functional magnetic resonance imaging (fMRI) data^45^. Linked standards for magnetoencephalographic (MEG)^48^ and EEG data^46^ have recently been published. See *bids-specification.readthedocs.io*/*en*/*stable*/*01-introduction.html* for an overview. To preserve detailed information about experimental events occurring during the data recording, BIDS uses the Hierarchical Event Descriptor (HED, version 2.0) system described at *HEDtags.org*^49,50^.

Datasets are available in.fdt format containing EEG, EMG, goniometer and event data in the same files. Multiple.fdt files are available for each subject representing multiple runs (blocks in one session) of the experiment. For a description of the files available for each run see Table 1 below. For a description of channel types see Table 2. For a full description of event markers see Online-only Table 1.

**Table 1:** Description of datasets.

File name	Description
sub-XXX_task-AudioCueWalkingStudy_run-XX_eeg.fdt	EEG, EOG, goniometer and event data
sub-XXX_task-AudioCueWalkingStudy_run-XX_eeg.set	Structure and dataset information on the above .fdt file
sub-XXX_task-AudioCueWalkingStudy_run-XX_channels.tsv	Channel type and channel labels
sub-XXX_task-AudioCueWalkingStudy_run-XX_coordsystem.json	EEG channel coordinate system
sub-XXX_task-AudioCueWalkingStudy_run-XX_electrodes.tsv	EEG channel locations
sub-XXX_task-AudioCueWalkingStudy_run-XX_eeg.json	Information on the recording software, filters used during recording, placement of reference and ground electrode etc.
sub-XXX_task-AudioCueWalkingStudy_run-XX_events.tsv	List of all events and event latencies contained in the dataset

**Table 2:** Description of channel types.

Channel numbers	Description
1–108	EEG data
109–112	EMG data - channels: 109–110 right tibialis anterior muscle; 111–112 left tibialis anterior muscle
113–115	Joint angle data - channels: 113 hip; 114 knee; 115 ankle

## Technical Validation

### EEG data

The EEG setup was carefully prepared to minimize potential artifacts and maximize data quality (see Data Acquisition above). For a basic estimation of data quality and validity of the experimental conditions, we compared the change of power in sensorimotor rhythms between standing and walking periods of the experiment. Event-related desynchronization (reduction in power, ERD) in the α and β bands during movement are typically smaller than during non-movement rest, as has been widely documented for upper^51^ and lower limb^52^ movements and during walking^25,33,34^.

The EEG data analysis for technical validation was performed on the data of 18 subjects using scripts written in MATLAB 2014a (The MathWorks) incorporating functions from EEGLAB 14.1.2^47^. The EEG data were high-pass filtered above 1 Hz (using a zero-phase FIR filter, order 7500) to minimize slow drifts, and low pass filtered below 200 Hz (using a zero-phase FIR filter, order 36). EEG channels with prominent artifacts were identified by visual inspection and removed. On average, 106 channels per participant (SD ± 2.2; range 102–108) were retained for analysis. The EEG data were then re-referenced to common average reference.

To perform automatic rejection of large movement-related artifacts, we applied artifact subspace reconstruction (ASR, in EEGLAB)^53,54^. Thresholds used for artifact rejection were conservative; the threshold for window rejection was disabled and the burst threshold was set to 20. The data were then segmented into step-locked epochs, from 1 s before to 3 s after each right heel strike during the uncued walking period. Epochs containing potential values exceeding ±3 SD of the mean were rejected. Because of the large number (>1000) of gait cycles per participant, from the uncued gait periods of the experiment we randomly selected 500 epochs from each participant’s data for further analysis.

**Fig. 2:**
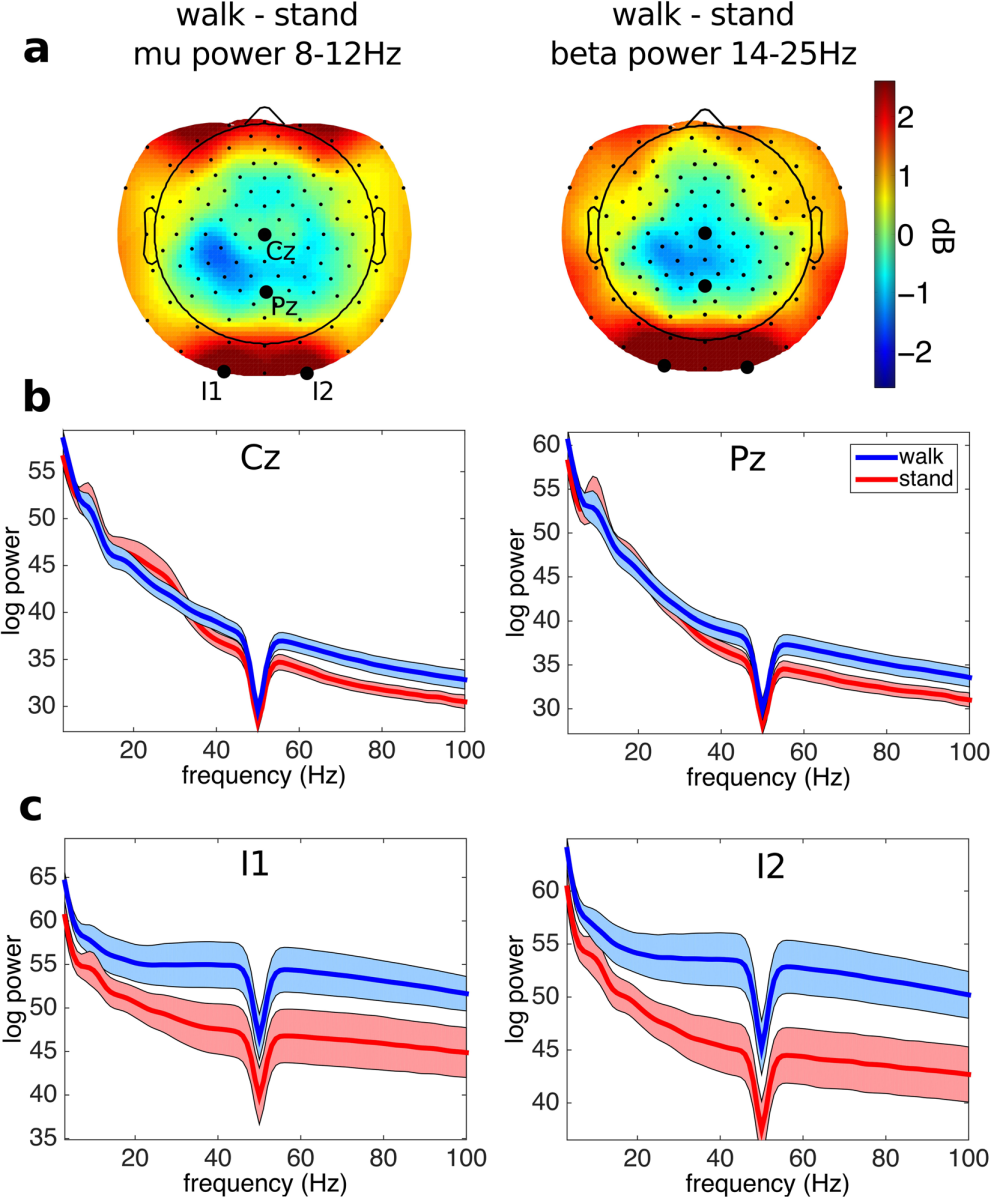
Technical assessment of the EEG data. (**a**) Scalp maps showing the scalp distribution of log power differences in mu and beta power between walking and standing periods. Cool colors represent negative differences, warm colors positive differences. Over central scalp, the maps show a clear reduction in power in the mu and beta bands during walking compared to standing. (**b**) Log power spectra for channels Cz and Pz during standing and walking. Mu and beta power is higher during standing (red trace) compared to walking (blue trace). Envelopes show ±3 standard errors of the mean. (**c**) Log power spectra for channels I1 and I2 during standing and walking. Power, especially at higher frequencies, is higher during walking (blue trace) compared to standing (red trace).

Differences in log spectral power between standing and walking were obtained for each channel. These differences were then averaged over subjects and average relative log power in the mu band (8–12 Hz) and beta band (14–25 Hz) were projected onto the scalp using the EEGLAB function *topoplot*. We also plotted the mean log spectral power for uncued walking and standing for electrode locations Cz, Pz, I1 and I2 (see Fig. 2). The expected smaller power of sensorimotor rhythms during movement compared to non-movement periods^25,33,34,51^ was also observed in these data. As shown in Fig. 2a,b, there is clearly less alpha and beta band power over the central scalp when the participants are walking compared to standing. By contrast, EEG power over lateral scalp areas was larger during movement than during standing, likely because of contributions from neck and facial muscle EMG during walking. At electrode locations Cz and Pz, power at higher frequencies (>30 Hz) during walking did not differ from standing (Fig. 2b,c), while at lateral electrode locations I1 and I2 during walking power at all frequencies was larger than during standing. Artifactual contamination of lateral electrode signals by neck muscle EMG during walking has been shown in previous studies^22,25,26^ and can be minimized by using blind source separation, typically Independent Component Analysis (ICA^55,56^) or frequency clustering^26^.

### Quality of foot switches and temporal precision of auditory cues

The quality of the foot switch data was essential to this experiment since the timing of auditory stimulation was adjusted online to match the pace of footsteps during the experiments. The foot switches were therefore continuously monitored during the whole experiment. If there were faulty activations, the treadmill and the paradigm was stopped so that the foot switches could be adjusted.

To make sure that we recorded the exact timing of the auditory cues we split the auditory output of the computer to feed the auditory cues into the digital input of the amplifier as described in the methods above. During post-processing, by subtracting the intended latencies of auditory cue onsets with the actual moments at which the cues sounded, we determined that there was a jitter in auditory cue timings of up to ±25 ms.

### EMG data

The quality of the EMG signals was assessed before beginning the experiment. Participants were instructed to repeatedly execute brisk foot dorsiflexion. During analysis, the EMG data were re-referenced to bipolar derivations, then high-pass filtered above 30 Hz (using a FIR filter, order 226), then rectified and low-pass filtered below 5 Hz (using a FIR filter, order 846) to obtain the signal envelope. The data were then segmented −1 to 3 s around right heel strikes during uncued walking. We then time warped the envelopes of the signal to the median step latency (across subjects) using linear interpolation. This procedure aligned the latencies of right and left heel strikes across trials.

**Fig. 3:**
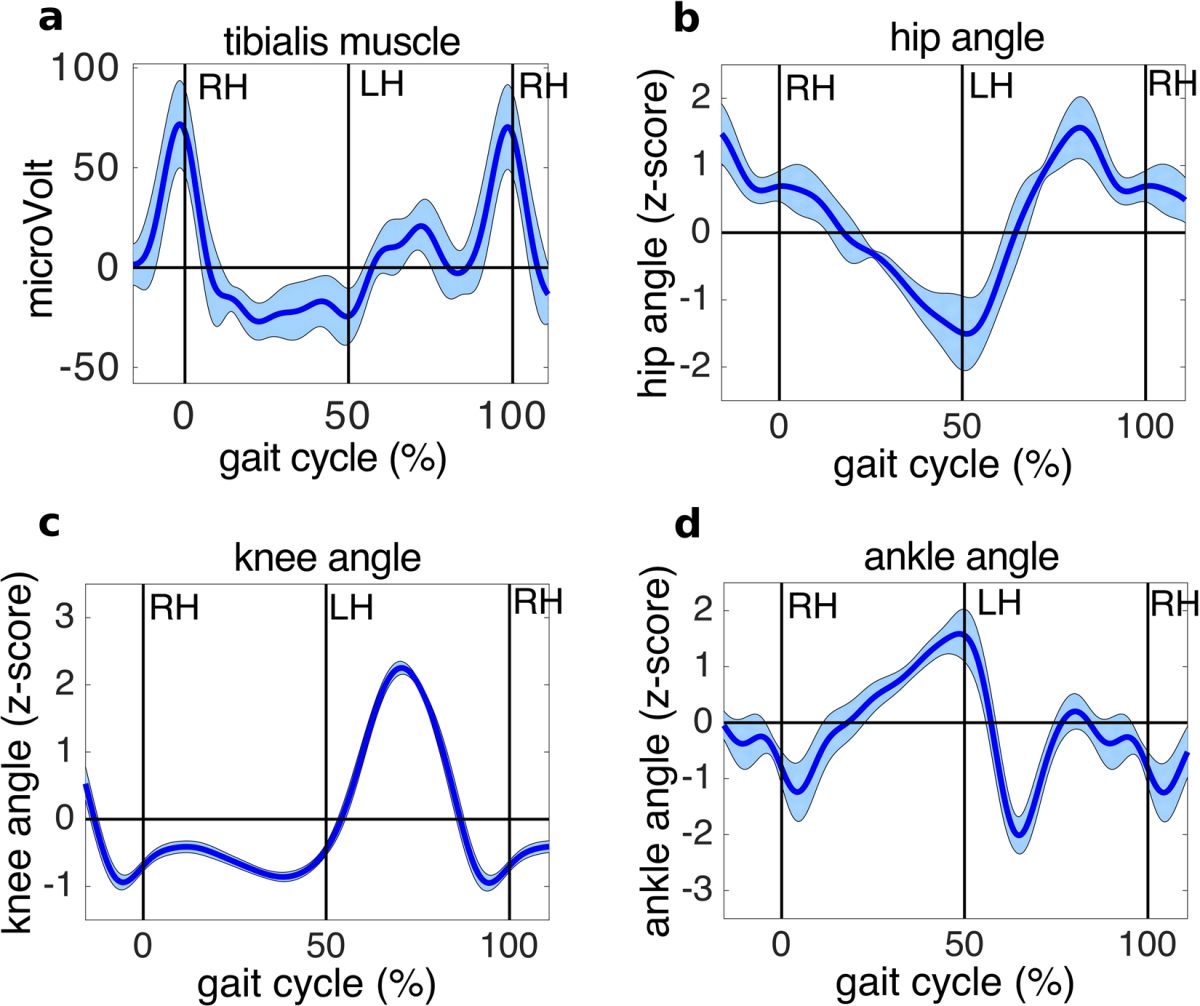
Technical assessment of the EMG and goniometric data. (**a**) Time course of EMG recorded from tibialis anterior muscle of the right leg. (**b**) Time course of right hip angle (**c**) Time course of right knee angle. (**d**) Time course of right ankle angle. EMG and goniometer data are time warped over one gait cycle. Envelopes show ±3 standard errors of the mean.

As shown in Fig. 3a, the muscle activations can be clearly seen during the walking condition; as expected, the right leg tibialis anterior is maximally active shortly before the right heel strike. The EMG recorded from the tibialis anterior muscle of the left leg was noisier and is not displayed.

### Goniometer data

The goniometers were carefully set up according to the manufacturer instructions (compare to^29^). After attaching the three goniometers to hip knee and ankle, the signals were checked while the participant walked on the treadmill. The goniometer signals were continuously monitored to ensure minimal interference and data corruption. To visualize the goniometer data, we high-pass filtered the data above 0.5 Hz (FIR filter order 3380) and then low-pass filtered the data below 5 Hz (FIR filter order 846). The data were then segmented (−1 to 3 s from right heel strikes) during uncued walking. We then time warped the goniometer data envelopes to the median step latency (across subjects) using linear interpolation. This procedure aligned time points of right and left heel strikes over trials. Sixteen of 18 subjects had usable goniometer data. Figure 3b–d shows the hip, knee, and ankle joint angles for 16 subjects during the uncued walking period on the treadmill.

## Usage Notes

Since EEG data recorded during walking are usually noisy, we recommend the use of advanced artifact rejection and artifact correction methods (available for example in the EEGLAB toolbox, freely available from sccn.ucsd.edu/eeglab/index.php^47^. For analysis of EEG data recorded during walking, see the following references:^22,24–26,31^. Other studies have looked at the types and morphologies of movement artifacts in EEG data during walking; these reports can be helpful to identify EEG artifacts recorded during MoBI experiments (see^57–59^).

## Data Availability

The code we developed to record EEG/EOG and goniometer data, run the paradigm and adapt cue rate online to the participants’ cadence in each trial are based on the TOBI SignalServer freely available online (*tools4bci.github.io/SignalServer)* a custom SimulinkModel running in MATLAB (The MathWorks) and a Ruby application for playing the auditory cues which is available upon request from the authors.

## Online-only Table

**Online-only Table 1:** Description of event markers.

Marker	Description
**uncued walking (trial start)**	
100	right heel strike
200	left heel strike
**preferred cadence walking**	
444	first auditory cue
114	first right cued step
151	auditory cues for right heel strike
251	auditory cues for left heel strike
101	right heel strike
201	left heel strike
**step advance tempo shifts (trial type I)**	
333	first auditory cue marking step advance tempo shift
113	first right step relative to tempo shift
153	auditory cues for right heel strike
253	auditory cues for left heel strike
103	right heel strike
203	left heel strike
**step delay tempo shifts (trial type II)**	
999	first auditory cue marking step delay tempo shift
119	first right step relative to tempo shift
159	auditory cues for right heel strike
253	auditory cues for left heel strike
109	right heel strike
209	left heel strike
**cued standing (separate block)**	
171	auditory cues previously associated with right heel strike
271	auditory cues previously associated with left heel strike
